# Nonlinear Dynamics Analysis of a Self-Organizing Recurrent Neural Network: Chaos Waning

**DOI:** 10.1371/journal.pone.0086962

**Published:** 2014-01-23

**Authors:** Jürgen Eser, Pengsheng Zheng, Jochen Triesch

**Affiliations:** Frankfurt Institute for Advanced Studies, Frankfurt am Main, Germany; Heidelberg University, Germany

## Abstract

Self-organization is thought to play an important role in structuring nervous systems. It frequently arises as a consequence of plasticity mechanisms in neural networks: connectivity determines network dynamics which in turn feed back on network structure through various forms of plasticity. Recently, self-organizing recurrent neural network models (SORNs) have been shown to learn non-trivial structure in their inputs and to reproduce the experimentally observed statistics and fluctuations of synaptic connection strengths in cortex and hippocampus. However, the dynamics in these networks and how they change with network evolution are still poorly understood. Here we investigate the degree of chaos in SORNs by studying how the networks' self-organization changes their response to small perturbations. We study the effect of perturbations to the excitatory-to-excitatory weight matrix on connection strengths and on unit activities. We find that the network dynamics, characterized by an estimate of the maximum Lyapunov exponent, becomes less chaotic during its self-organization, developing into a regime where only few perturbations become amplified. We also find that due to the mixing of discrete and (quasi-)continuous variables in SORNs, small perturbations to the synaptic weights may become amplified only after a substantial delay, a phenomenon we propose to call *deferred chaos*.

## Introduction

A fundamental question in Neuroscience is how cortical circuits acquire the structure required to perform desired computations. A range of different plasticity mechanisms shape neural circuits, but their interaction at the network level remains poorly understood. Recent modeling work has shown that recurrent spiking neural networks with multiple forms of plasticity can learn interesting representations of sensory inputs [1] and reproduce experimental data on the statistics and fluctuations of synaptic connection strengths in cortex and hippocampus [2]. These self-organizing recurrent networks (SORNs) rely on an interplay of spike-timing dependent plasticity (STDP) and different homeostatic mechanisms. While these networks offer a plausible explanation for the experimentally observed approximately log-normal distribution of excitatory synaptic efficacies, their dynamics are still poorly understood. Here we try to shed light on this issue by employing tools from nonlinear dynamics analysis. Specifically, we perform a perturbation analysis, where we investigate how the evolution of a network changes in response to a small perturbation. We characterize the degree of chaos by an estimate of the maximum Lyapunov exponent and study it at different stages of network evolution.

Several previous studies have investigated the occurrence of chaos and associated irregular dynamics [3] in discrete and continuous time artificial neural networks with symmetric oscillators [4], asymmetric weight structures [5] and both weak and full connectivity [6]–[9]. Most studies have used sigmoidal transfer functions like the hyperbolic tangent [10]–[12]. The high degree of abstraction of such models often makes it hard to directly relate them to brain function, e.g. see [13]. Robust chaos is common in such systems and with more units in the network an increasing number of positive Lyapunov exponents has been observed [14], [15]. The tendency for chaotic dynamics in networks with excitatory and inhibitory neurons can be reduced by Hebbian learning mechanisms as well as certain external stimuli [10], [16]. Self-organization due to Hebbian learning is frequently assumed to be the responsible mechanism for the observed stabilization [10], [17]. Interestingly, it has also been argued that recurrent neural networks should operate at the edge of chaos in order to maximize their computational power [18]–[20]. This suggests that neither highly chaotic dynamics as prevalent in random networks nor strongly regular dynamics may be desirable for the brain. Here we investigate the degree of chaos in the SORN model as studied in [2]. An interesting property of these networks is their hybrid nature that mixes discrete and (quasi-)continuous variables [21]. Our major finding is an overall reduction of chaos during its self-organization, with the network settling into a regime where only few perturbations become amplified. Due to the mixing of discrete and (quasi-)continuous variables in SORNs, they are instances of hybrid dynamical systems [21]. We also show that because of their hybrid nature, small perturbations to the synaptic weights may become amplified only after a substantial delay, a phenomenon we propose to call *deferred chaos*.

## Methods

### Network Model

The network model is almost identical to the one in [2]. It consists of 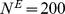 excitatory and 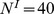 inhibitory threshold neurons linked through weighted synaptic connections. 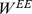 represents the connections between excitatory neurons. Initial connectivity is sparse with a connection probability of 0.1, but can change during network evolution. Self-connections of the excitatory neurons are forbidden. 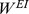 are the inhibitory-to-excitatory connections with connection probabilities of 0.2. 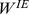 denotes excitatory-to-inhibitory connections which are all-to-all and remain fixed at their random initial values. Connections between inhibitory neurons were left out to keep the model simple and to stay consistent with the previous work on these networks [1], [2]. Unless specified otherwise, all initial weights are drawn from a uniform distribution over the interval 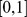 and then normalized such that the sum of weights projecting to a neuron is one.

The binary variables 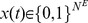 and 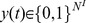 represent the activity of the excitatory and inhibitory neurons at a discrete time 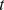, respectively. The network activity state at time step 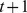 is given by: 
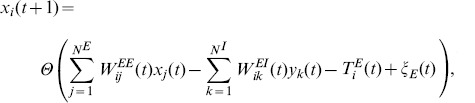
(1)

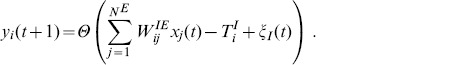
(2)

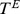
 and 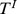 denote threshold values for the excitatory and inhibitory neurons, respectively. Initially, they are uniformly distributed in the interval 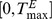 and 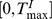, but not bounded during network evolution. 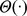 is the Heaviside step function. 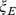 and 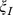 represent Gaussian white noise with 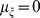 and 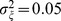.

The connection strength of nonzero weights between excitatory neurons is subject to the following spike-timing dependent plasticity (STDP) rule: 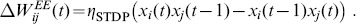
(3)


If 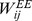 becomes negative due to this update, the synapse is immediately eliminated.

At every time step we normalize all incoming weights to an excitatory neuron by 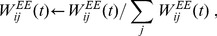
(4)

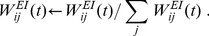
(5)


Furthermore, the applied intrinsic plasticity (IP) mechanism regulates the spiking thresholds in such a way that all neurons exhibit the same average firing rate 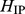: 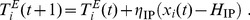
(6)


Thresholds of inhibitory neurons remain at their initial values.

Inhibitory weights are subject to a different STDP rule. We implement a form of inhibitory spike-timing dependent plasticity (iSTDP) for the 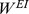 connections. If an inhibitory neuron is active and the excitatory neuron receiving input from this inhibitory neuron stays silent in the following time step, the corresponding weight will be weakened by the amount 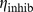. If, however, the excitatory neuron is active despite receiving input from the inhibitory unit, then the weight will be strengthened by an amount 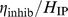: 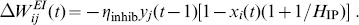
(7)


This rule aims to balance excitation and inhibition and is inspired by [22]. If an inhibitory neuron succeeds in silencing a target excitatory cell with its spike, the inhibitory influence is weakened. Conversely, if the inhibitory neuron fails to silence the excitatory cell, its inhibitory influence is strengthened.

We also introduce a structural plasticity (SP) mechanism to compensate for the synapse elimination induced by STDP. We conceive this rule as a Bernoulli experiment for each neuron. At every time step there is a small probability 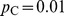 that a neuron creates new synapses to other excitatory cells. The number of connections is drawn from a Poisson distribution with mean 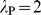 and their weight is set to 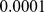. The parameters used in the simulations were as follows: 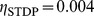, 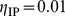, 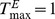, 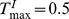, 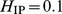, 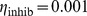.

### Perturbation analysis

We apply perturbation analysis to measure the stability of the network during different stages of its evolution as observed in [2]. The network has a finite set of elements, each of which takes a discrete (unit activations) or (quasi-)continuous (weights and thresholds) value. To perform the analysis, we simulate a first network (the *original*) up to a certain time step. Then a *copy* of the network is made and a small perturbation is applied to this copy. Both original network and copy are simulated with identical noise as well as under the same influence of structural plasticity and we compare their subsequent evolution. To this end, we consider the Euclidean or Hamming distance of network weights, thresholds and activities, respectively. To construct a “baseline” Euclidean distance for the purpose of normalization due to changing statistics during network evolution, we consider the current weight matrices, threshold and input vectors of the unperturbed SORN and compute their Euclidean distance to 100 random matrices and vectors, respectively. These are generated by randomly permuting the rows/entries of the original matrix/vector. This keeps the statistics of the weights, thresholds and inputs identical. The average distance obtained in this procedure then serves as the normalization factor 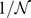 when calculating the Euclidean distances. An analogous normalization for the Hamming distances is not necessary, however. As stated above, the implemented IP mechanism guarantees a constant average firing rate. Perturbations are performed by strengthening or newly generating one randomly selected entry of the 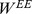 weight matrix by an amount proportional to 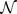. Here, 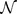 corresponds to the inverse normalization factor of the 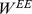 matrix. The perturbation strength is set to 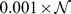.

Note that a perturbation of the network weights will also affect the unit activities, because an increased (or decreased) synaptic weight may allow (or prevent) a neuron from reaching its firing threshold in a certain situation. In fact, since the dynamics of the weights of the network depends exclusively on the synaptic normalization and the unit activities via the STDP mechanism, a small change to one weight can only percolate to other weights after it has resulted in an effect on the discrete unit activities. Conversely, a change of unit activities will immediately affect the synaptic weights, because the firing (or silence) of a neuron may lead to the triggering (or not) of the STDP mechanism which adapts the synaptic weights. Note that the effects of perturbations to unit activities will generally be bigger, because an extra spike in a unit can lead to changes in all incoming and all outgoing connections of this neuron at the same time.

Because perturbations to the weights will only affect unit activities after a delay (at least one time step later), we define two different Lyapunov exponents. The first, immediate Lyapunov exponent 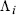 quantifies any amplifications of perturbations to the weight matrix following the perturbation as long as the neuron activities remain unaffected. The second, delayed Lyapunov exponent 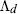 quantifies amplifications of perturbations to the weight matrix after these have altered the neuron firing patterns. Specifically, let 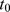 be the time of a perturbation which we observe for a total of 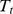 time steps and 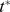 be the time step right before the unit activities start differing between the perturbed and unperturbed networks (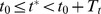). We estimate 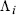 according to: 
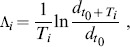
(8)where 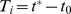 and 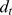 denotes the normalized Euclidean distance corresponding to the excitatory-to-excitatory weight matrix. Analogously, we estimate 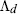 as: 
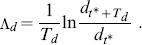
(9)

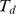
 is set to 10 time steps. The combined maximum Lyapunov exponent 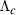 is computed as follows: 
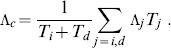
(10)


If the activities are not affected at all within the investigated time period or if they are influenced immediately after the perturbation, the combined Lyapunov exponent simply reads 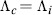 with 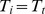 and 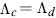, respectively. Else if 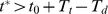, we used 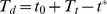. Perturbations which completely vanish are ignored.

To characterize network dynamics in different stages of network evolution, perturbations are performed at different time points. At each stage, we consider 10 independent networks. Each network is considered at 10 different time points within 100 time steps during this phase. For each time step we perform 10 perturbations. Thus, we perform a total of 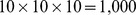 perturbations for each stage of the network's evolution.

## Results

**Figure 1:**
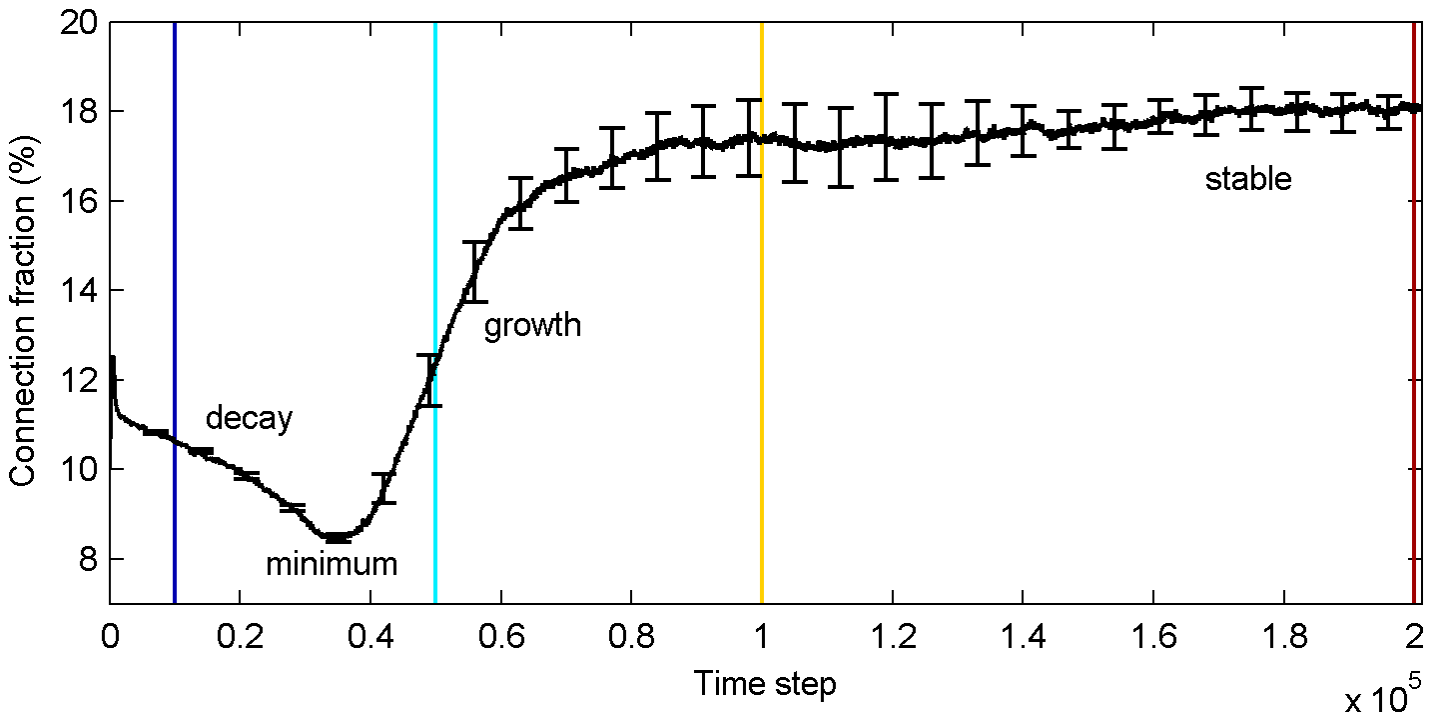
Average fraction of nonzero excitatory-to-excitatory connections as a function of time. The different phases of connectivity and time periods of investigation are indicated in terms of color. Errorbars represent standard errors (10 simulations) and are given every 7,000th time step.

As the network develops, its connectivity changes due to the action of the different plasticity mechanisms. Zheng et al. [2] observed that the network goes through different phases as indicated by the number of excitatory-to-excitatory connections present in the network. Fig. 1 shows the average fraction of excitatory-to-excitatory connections present in the network as a function of time. During an initial decay phase (around time step 10,000), many connections are driven to zero strength and are removed from the network. The connectivity becomes minimal between time step 30,000 and 40,000. In the subsequent growth phase, connectivity increases again. Around time step 100,000 the network has entered the stable phase where connectivity stays roughly constant.

### Effect of perturbations on network weights and thresholds

**Figure 2:**
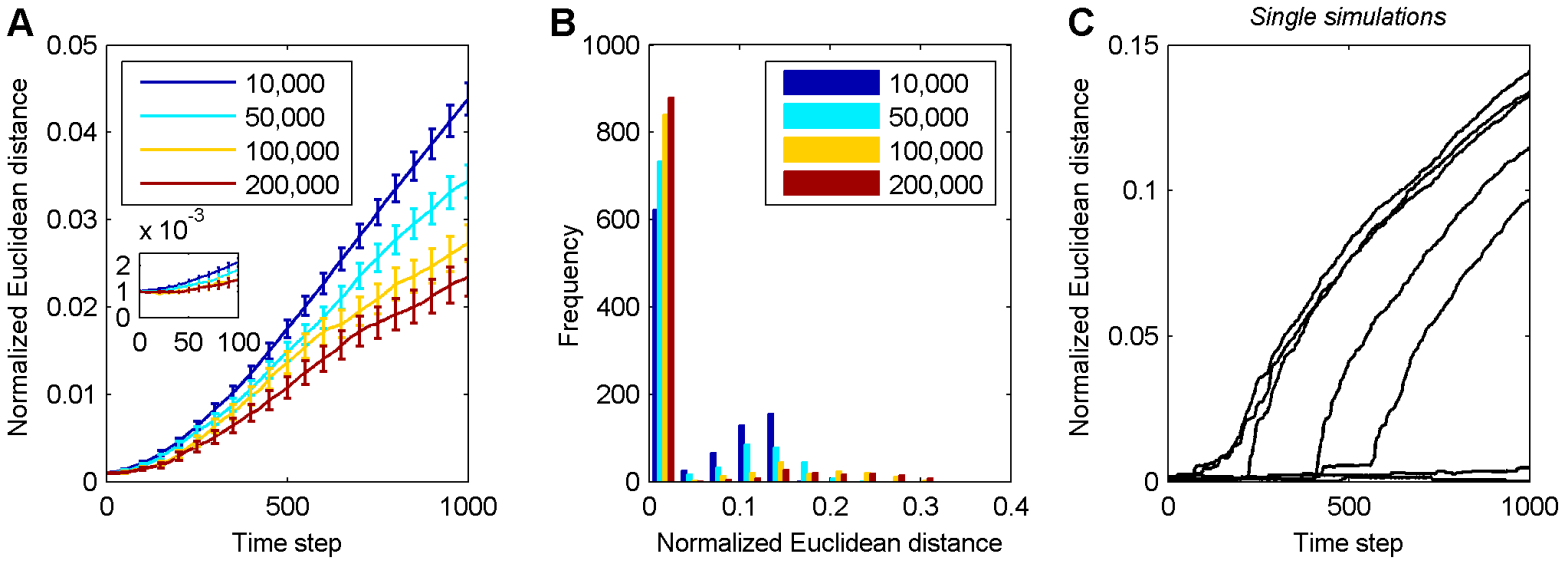
Long term development of normalized Euclidean distances between excitatory weight matrices of perturbed and unperturbed networks. **A** Averaged result over 1,000 simulations with error bars indicating standard errors of the mean. The inset shows a magnification of the initial 100 time steps. **B** Histogram of normalized Euclidean distances at 1,000th time step in A. In figures A–B, the color indicates the starting time of the perturbations as shown in the legend. **C** Examples of single simulations in the decay phase: the normalized Euclidean distance often stays at a low value for several hundred time steps but then displays an extensive amplification.

We measured the long term development of the normalized Euclidean distance between the excitatory-to-excitatory weight matrix of the original and the perturbed network over 1,000 time steps after the perturbation as described in the Methods. This was done for SORNs in the three different phases of their development: starting at 10,000 time steps (decay phase), 50,000 time steps (growth phase), 100,000 and 200,000 time steps (early and late in their stable phase). The results are shown in Fig. 2 **A**. The average normalized Euclidean distance tends to increase with time. This increase is fastest for networks in the decay phase and slowest for networks in the stable phase. Fig. 2 **B** shows a histogram of normalized Euclidean distances between the original and perturbed network 1,000 time steps after the perturbation. Note that a majority of perturbations are not strongly amplified (normalized Euclidean distance stays close to zero) and only few perturbations have a strong effect on the network. During the decay phase around time step 10,000 (blue) there are many more such perturbations producing a strong effect compared to the other phases. In the first bin, the height of color bars increases monotonically as the networks go from decay to stable phase, which indicates that they become less sensitive to perturbations with time. Fig. 2 **C** gives examples of how individual perturbations during the decay phase of the network develop. The normalized Euclidean distance between the weight matrix of the original and the perturbed network tends to maintain a small value but may suddenly increase after several hundred time steps.

**Figure 3:**
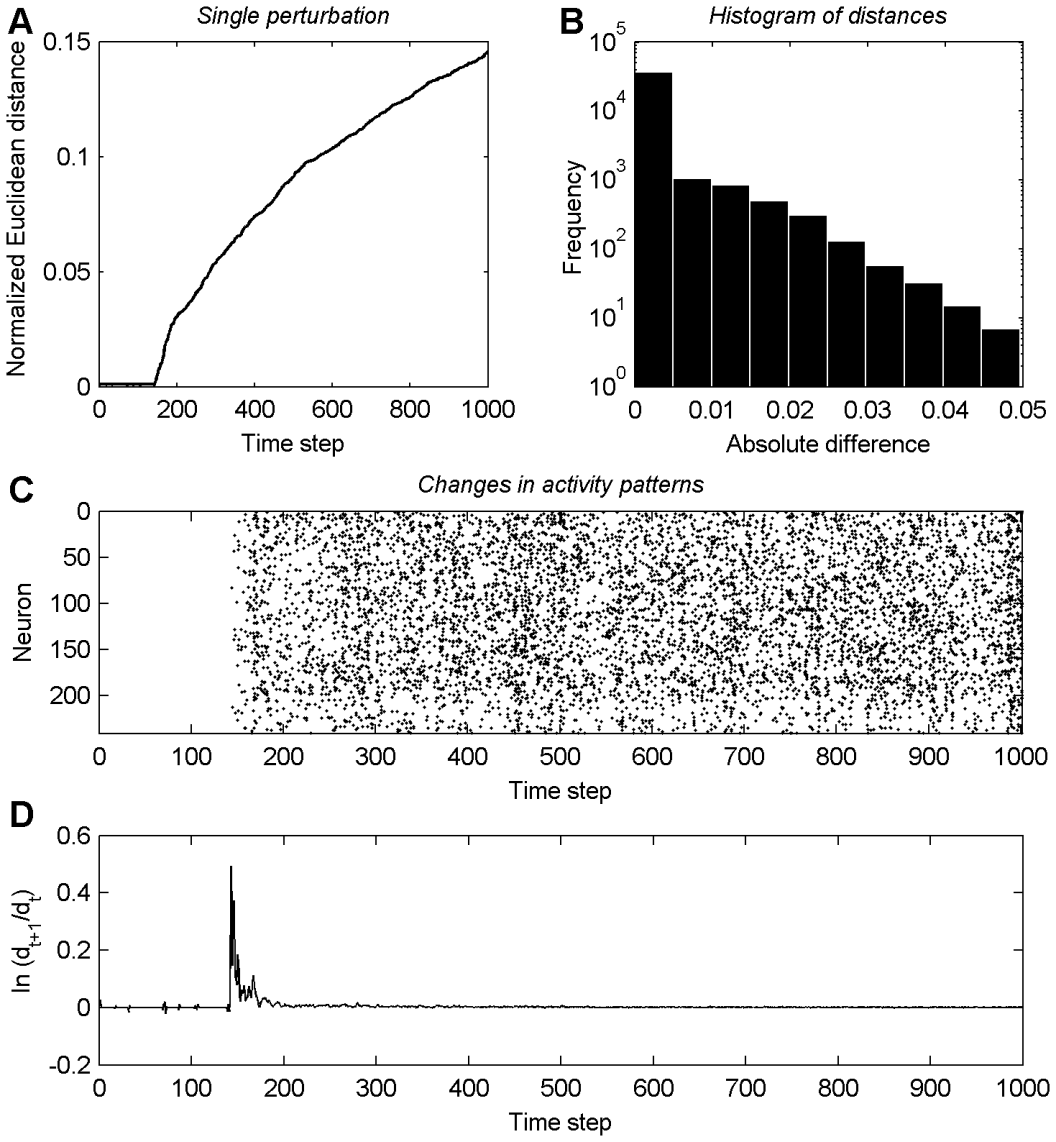
Example of a single amplified perturbation. **A** Development of the normalized Euclidean distance in a single perturbation during the decay phase. **B** Histogram of the single absolute differences of the excitatory-to-excitatory weights at time step 1,000 in A. **C** Differences in neuron activity patterns between the perturbed and the unperturbed network. **D** Time course of 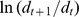. 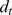 corresponds to the normalized Euclidean distance in A at time step 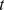.

To illustrate this effect more clearly, we show as an example the changes of the excitatory-to-excitatory weights, the activity patterns and the time development of 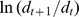 in a single amplified perturbation from the decay phase in Fig. 3. Fig. 3 **A** displays the time course of the Euclidean distance between the weight matrices of the perturbed and unperturbed networks. Note the sharp increase around time step 150. Fig. 3 **B** shows a histogram of the single absolute differences of the excitatory-to-excitatory weights at the 1,000th time step in **A**. Interestingly, only a comparatively small fraction of weights contribute noticeably to the total normalized Euclidean distance and most of the connection strengths remain close to their original values. Data points in Fig. 3 **C** indicate situations where a neuron stays silent in the perturbed network and is active in the unperturbed network or vice versa. It is apparent that the sudden increase of the normalized Euclidean distance between both networks around time step 150 in **A** coincides both with upcoming differences in the activity patterns in **C** and an underlying time period of positive values of 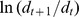 in **D**.

The observed delayed amplification of the perturbation to the synaptic weights is due to the mixing of discrete and (quasi-)continuous variables in the network. The perturbation of a weight can only percolate to other weights after it has managed to alter one or more of the discrete unit activities. Due to the threshold function in the units' dynamics and depending on the size of the weight perturbation, this may take a substantial amount of time. We propose to call this phenomenon *deferred chaos*. Note that the increase in the Euclidean distance following changes in the unit activities in Fig. 3 **A** is not exponential. This also holds for the examples in Fig. 2 **C**. The reason for this is again that the changes to the synaptic weights are mediated by changes to the unit activities. At any time step, only a small number of weights are affected via the STDP mechanism and each of them will change its value by 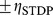. Thus the total amount of weight change per time step is limited, thereby preventing an extended exponential growth.

**Figure 4:**
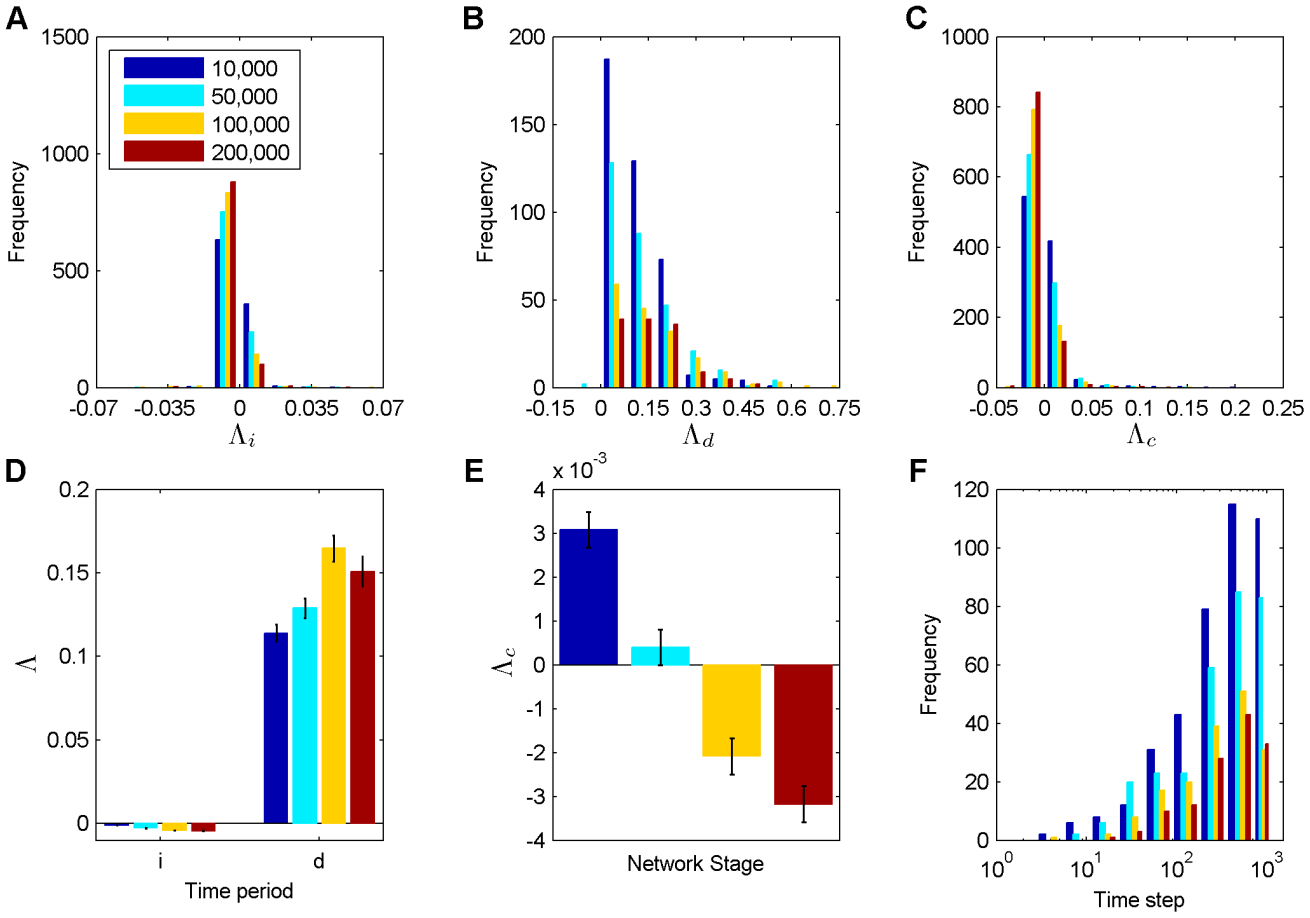
Deferred chaos. **A** Histogram of the immediate Lyapunov exponent. The legend shows the starting times of perturbations for each subfigure. **B** Histogram of the delayed Lyapunov exponent. **C** Histogram of the combined Lyapunov exponent. **D** Time course of the immediate and the delayed Lyapunov exponent. **E** Time course of the combined Lyapunov exponent. **F** Histogram of the time a weight perturbation needs to affect the neuron activities. The data correspond to Fig. 2. Errorbars in D and E indicate standard errors of the mean.

To quantify the *deferred chaos*, we considered the immediate, delayed and combined Lyapunov exponents introduced above. Fig. 4 **A**–**C** show histograms of 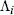, 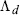 and 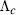 at different stages of network evolution. The majority of perturbations produce a negative value of 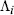 across all phases. We also find more perturbations that have a slightly positive value during the decay phase (around time step 10,000) than in any other phase. Especially, the stable phase (around time steps 100,000 and 200,000) displays a huge amount of slightly negative values. Fig. 4 **B** shows histograms of estimated 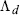 values for the different phases of network evolution. Almost all estimates are positive, indicating chaotic behavior right after a perturbation has affected the unit activities. Note that only a fraction of perturbations ever affect the unit activities, however. This fraction is highest in the decay phase around time step 10,000 and becomes smaller with network evolution (10,000: 40.6%, 50,000: 30.1%, 100,000: 16.9%, 200,000: 13.0%). Also note that since the growth of the Euclidean distance of the weight matrix after “take-off” is generally not exponential as explained above (compare Fig. 3 **A**), the estimate of 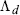 depends on the time interval 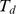 over which it is estimated. Results are shown for 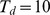 time steps (compare eq. (9)). Longer time intervals will lead to a smaller estimate of 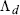. Fig. 4 **C** shows histograms of 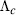 values for the different phases of network evolution, which are estimated by combining 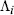 and 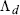 according to (10). The number of negative 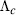 estimates is smallest for networks in the decay phase and highest for networks in the stable phase.

Fig. 4 **D**, **E** show the average 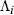, 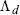, and 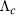 for the four different stages of network evolution. 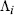, 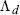 and 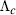 are significantly different from zero across all phases (except 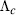 during the growth phase: 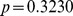, else: 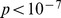; t-test). On average, 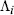 (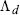) is strictly negative (positive) during the whole network evolution. 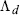 significantly increases from growth to stable phase. However, we also find a significant decrease of 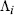 and 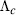 from decay to stable phase (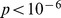 for 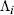, 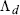 and 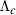; ANOVA with multiple comparisons and post-hoc t-test), indicating chaos waning. Specifically, 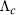 starts at a positive value during the decay phase, crosses the critical line and settles down to negative values when entering the stable phase. Finally, we find a significant increase according to 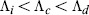 averaged across all stages of network evolution (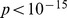; ANOVA with multiple comparisons and post-hoc t-test). Only 16 out of a total of 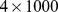 perturbations vanished completely and were excluded, which should not strongly bias the results.

We also quantified the distribution of time periods of how long it takes a weight perturbation to influence the neuron activities. Fig. 4 **F** shows histograms of such “take-off” times depending on the stage of network evolution. Across all network stages the perturbations typically need several hundred time steps to alter activities for the first time. Note that the relatively steady increase of the normalized Euclidean distance in Fig. 2 **A** results from averaging many curves like the ones in Fig. 2 **C** with different “take-off” times.

**Figure 5:**
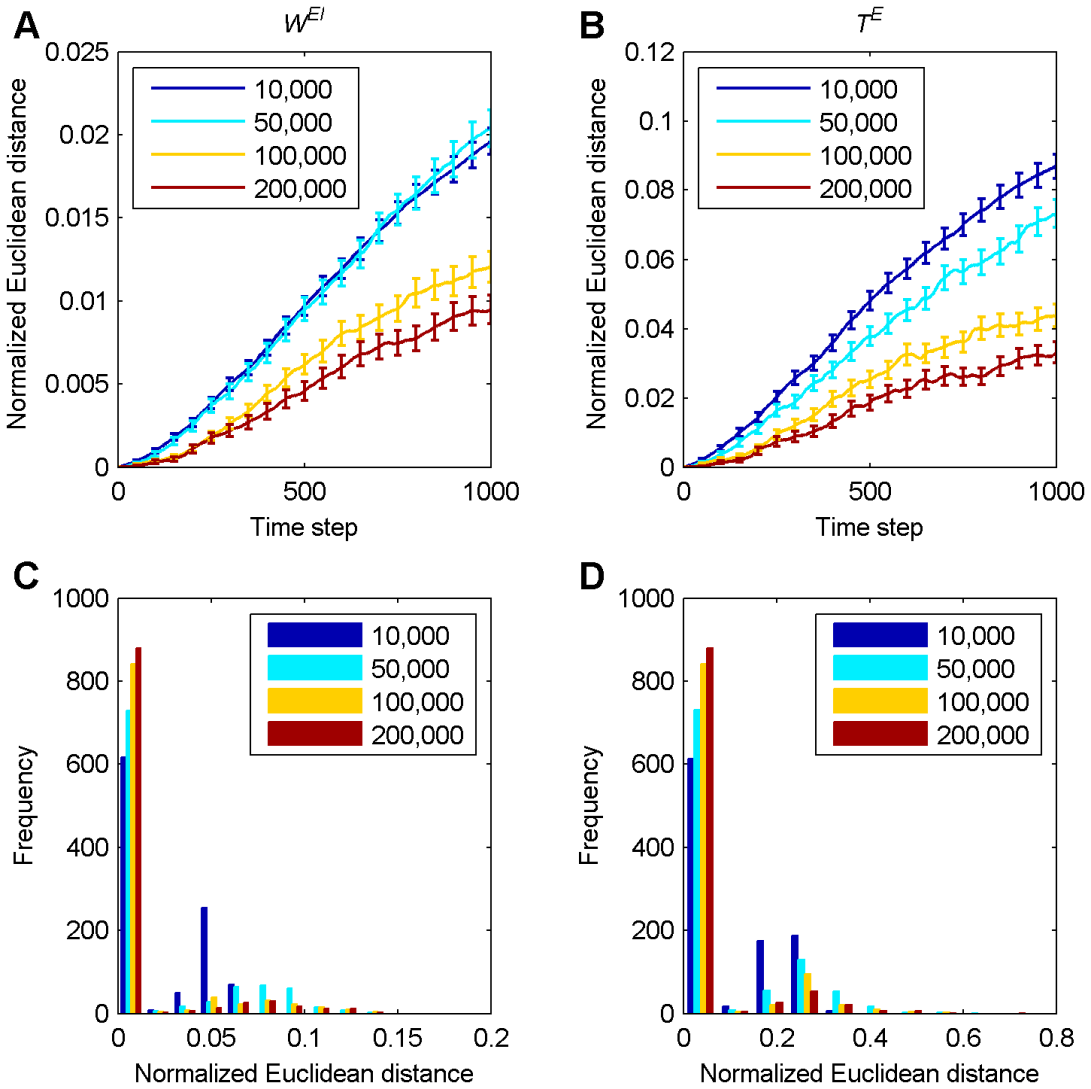
Long term development of normalized Euclidean distances between inhibitory-to-excitatory weight matrices and excitatory threshold vectors. The color indicates the starting time of the perturbations as shown in the legend. **A** Averaged normalized Euclidean distance between inhibitory-to-excitatory weight matrices over 1,000 simulations for 1,000 time steps. **B** Averaged normalized Euclidean distance between excitatory threshold vectors over 1,000 simulations for 1,000 time steps. Errorbars indicate standard errors of the mean in A and B. **C** Histogram of normalized Euclidean distances at 1,000th time step in A. **D** Histogram of normalized Euclidean distances at 1,000th time step in B.

Fig. 5 shows how a perturbation to the excitatory-to-excitatory weights affects inhibitory-to-excitatory connections and excitatory thresholds. Again, perturbations are amplified least when the network has reached the stable phase around time step 100,000 and 200,000. In Fig. 5 **A**, there is no significant difference between the decay phase (around time step 10,000) and the growth phase (around time step 50,000), which is in contrast to the development of the excitatory-to-excitatory weights as shown in Fig. 2 **A** and the thresholds as shown in Fig. 5 **B**. However, part **C** and **D** of Fig. 5 exhibit the same stabilizing phenomenon as Fig. 2 **B**: the fraction of perturbations that are not strongly amplified increases monotonically across the different stages of network evolution.

### Effect of perturbations on the unit activities

**Figure 6:**
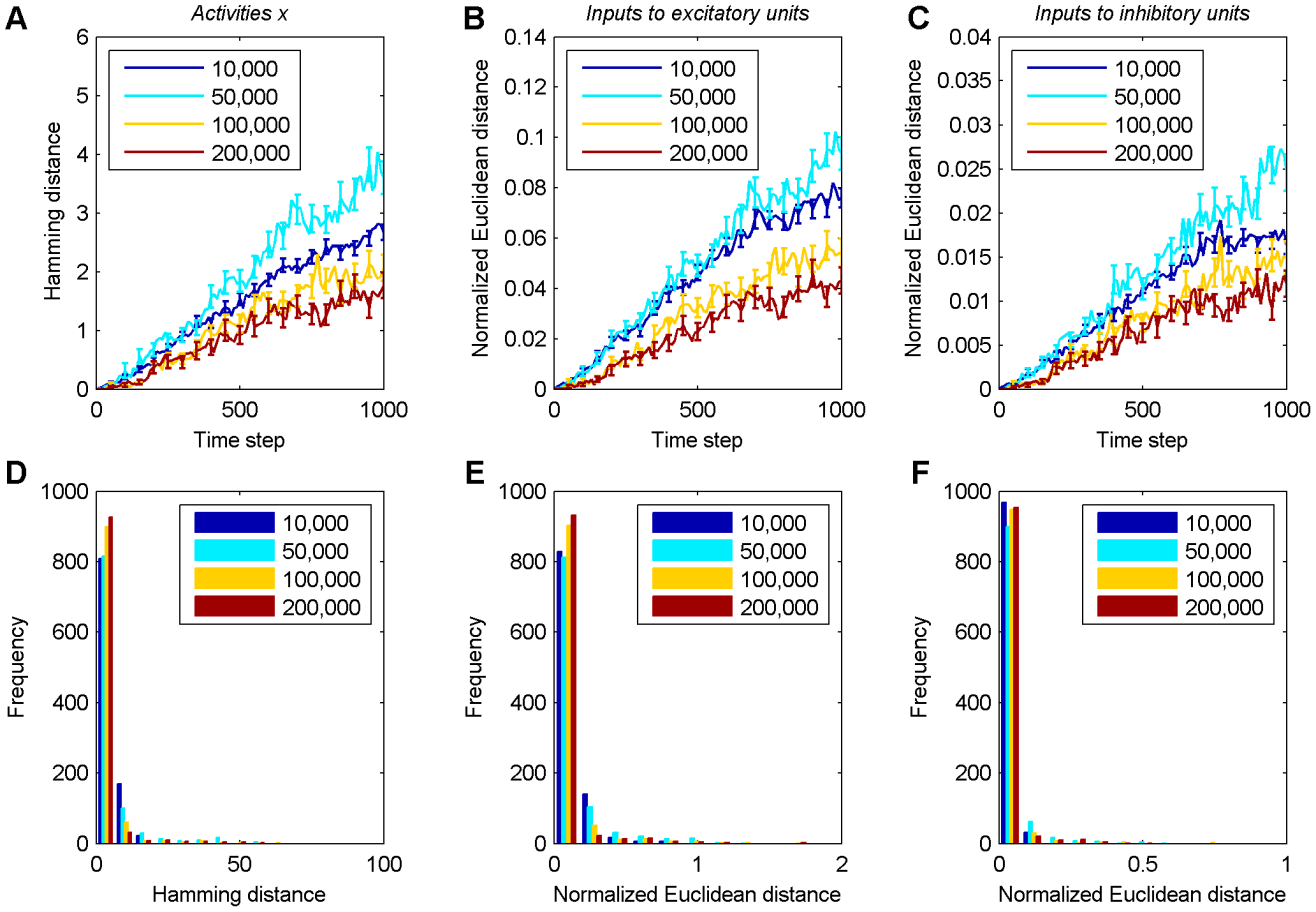
Perturbation analysis of neuron activities. The legend shows the starting times of perturbations. **A** Averaged Hamming distance between neuron activities of the perturbed and unperturbed networks over 1,000 simulations for 1,000 time steps. Only every tenth time step is plotted. Error bars indicate standard errors of the mean in A–C and are given every 50th time step. **B** Averaged normalized Euclidean distance of excitatory neuron inputs over 1,000 simulations for 1,000 time steps. **C** Averaged normalized Euclidean distance of inhibitory neuron inputs over 1,000 simulations for 1,000 time steps. **D**–**F** Histograms of Hamming distances and normalized Euclidean distances at 1,000th time step in A–C, respectively.

In Fig. 3 **C** we already saw how a single perturbation to the synaptic weights can influence the unit activities. To study this effect more systematically, we consider the evolution of the Hamming distance between the activities of the excitatory units in the original and the perturbed network. Fig. 6 **A** shows that the average long term development of the Hamming distance increases with time during all phases. Consistent with the results from the previous section, the amplification of an initial perturbation tends to become smaller as the network develops. In the stable phase, perturbations grow most slowly. A similar behavior is observed for the non-discrete inputs to the excitatory and inhibitory units. Specifically, we consider the normalized Euclidean distance between the arguments of the Heaviside step functions in the update equations (1) and (2) for the excitatory (Fig. 6 **B**) and inhibitory units (Fig. 6 **C**). Note that the effects of the perturbation are generally small. A mean Hamming distance of 4.5 after 1,000 time steps (compare Fig. 6 **A**) means that on average only four or five units out of 200 have a different activity state. Often the perturbation is immediately “forgotten” in the next time step and only infrequently it is amplified up to a limiting mean value of 36 corresponding to two independent networks with sparse activity of 10% active units per time step. Nevertheless, we find once again in two out of three cases the smallest amount of strongly amplified simulations in the stable phase, which is evidence for the network's stabilization (compare Fig. 6 **D**–**F**).

**Figure 7:**
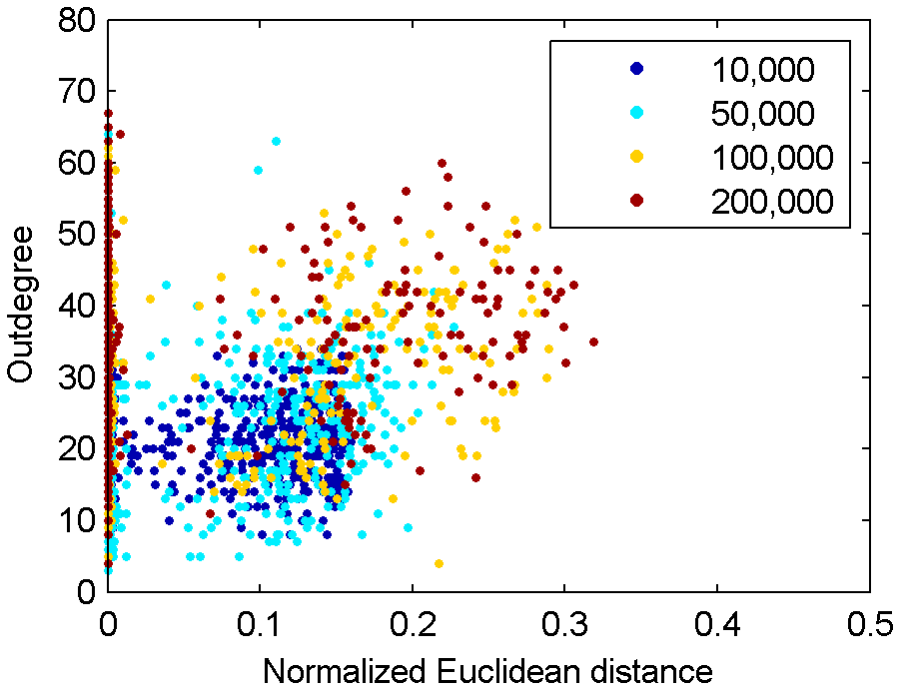
Relationship of outdegree vs. normalized Euclidean distance at 1,000th time step in Fig. 2 A. Scatter plot of the perturbed neurons' outdegrees and the corresponding normalized Euclidean distances between the excitatory weight matrices. Coefficients of correlation between the outdegree and the normalized Euclidean distance are not significantly different from zero.

We speculated that the effect of a perturbation to a synaptic weight may depend on the outdegree of the receiving neuron. A unit with a high outdegree connects to many other units in the network. Therefore, altering one of its incoming weights may affect a large part of the network. This raises the question if the normalized Euclidean distance is correlated to the outdegree of the neuron that receives the perturbed weight. This is not the case, however. Fig. 7 shows a scatter plot of the perturbed neuron's outdegree vs. the normalized Euclidean distance between excitatory weight matrices of original and perturbed network after 1,000 time steps for 1,000 perturbations in each of the three phases of network development. Coefficients of correlation 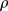 are small and not significant at the 5% level in any of the four stages (10,000 time steps: 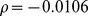, 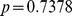; 50,000 time steps: 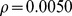, 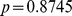; 100,000 time steps: 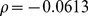, 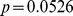; 200,000 time steps: 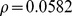, 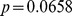; two-tailed t-test).

## Discussion

The potential role of chaos in the brain has long been a topic of debate. On the one hand, recent work suggests that chaotic dynamics could play a central role in approximating stochastic inference schemes, e.g., [23]. On the other hand, recent theoretical considerations and experimental data support the view that the brain operates in a regime close to criticality that is neither highly chaotic nor strongly regular [18]–[20], [24]. A recent analysis by Priesemann et al. indicates that the human brain may in fact favor a slightly subcritical operating regime across different vigilance states [25]. This raises the question how the brain tunes its dynamics towards such a slightly subcritical regime.

Here we have investigated the dynamics of the recently proposed self-organizing recurrent neural network model (SORN) [1], [2]. This model has been shown to learn effective representations of dynamic inputs and to reproduce experimental data on the statistics and fluctuations of synaptic connection strengths in cortex and hippocampus. Here we have studied the network's self-organization without any structured input but under the influence of weak noise. To characterize the network's dynamics during different phases of its self-organization, we performed a perturbation analysis and quantified the sensitivity of the network to small changes to its synaptic weights. Our major findings are that only a fraction of perturbations becomes amplified within a limited time window, and this fraction decreases across the different phases of network self-organization. This is consistent with findings in other recurrent network architectures with Hebbian-like plasticity mechanisms showing a development towards more stable dynamics [10], [14], [26]. Interestingly, most perturbations to the synaptic weights remained at a “critical” level or even disappeared again. We showed that amplification of a perturbation depends on whether it affects the neural activity or not. Perturbations that are amplified produce drastic changes in the network's firing patterns compared to the cases where neuron activity in perturbed and unperturbed network stays identical. Importantly, we found that such changes in the network's activity patterns could occur after substantial delays, a phenomenon we refer to as *deferred chaos*. This effect is a consequence of the hybrid nature of our network in that it mixes discrete (neuron activities) and (quasi-)continuous (weights and thresholds) variables [21]. Since changes to the synaptic weights are driven by the neurons' activity patterns, a change to a synaptic connection has to first affect the activitiy of the recipient neuron before it can percolate to other weights in the network. Depending on the size of the initial perturbation this can take a very long time across all different phases of network self-organization. It is an open question whether there are interesting engineering applications for such *deferred chaos*.

Overall, our results are broadly consistent with earlier findings that similar networks may develop dynamics that are close to the critical regime [26]. Further analysis is needed to understand how the network's dynamics is influenced by structured inputs.
